# Space-efficient representation of genomic k-mer count tables

**DOI:** 10.1186/s13015-022-00212-0

**Published:** 2022-03-21

**Authors:** Yoshihiro Shibuya, Djamal Belazzougui, Gregory Kucherov

**Affiliations:** 1 LIGM, Université Gustave Eiffel, Marne-la-Vallée, France; 2 CAPA, DTISI, Centre de Recherche sur l’Information Scientifique et Technique, Algiers, DZ Algeria; 3 Skolkovo Institute of Science and Technology, Moscow, Russia

**Keywords:** *k*-mers, Counts, Compression, Compressed static function, Bloom filter

## Abstract

**Motivation:**

*k*-mer counting is a common task in bioinformatic pipelines, with many dedicated tools available. Many of these tools produce in output *k*-mer count tables containing both *k*-mers and counts, easily reaching tens of GB. Furthermore, such tables do not support efficient random-access queries in general.

**Results:**

In this work, we design an efficient representation of *k*-mer count tables supporting fast random-access queries. We propose to apply Compressed Static Functions (CSFs), with space proportional to the empirical zero-order entropy of the counts. For very skewed distributions, like those of *k*-mer counts in whole genomes, the only currently available implementation of CSFs does not provide a compact enough representation. By adding a Bloom filter to a CSF we obtain a Bloom-enhanced CSF (BCSF) effectively overcoming this limitation. Furthermore, by combining BCSFs with minimizer-based bucketing of *k*-mers, we build even smaller representations breaking the empirical entropy lower bound, for large enough *k*. We also extend these representations to the approximate case, gaining additional space. We experimentally validate these techniques on *k*-mer count tables of whole genomes (*E. Coli* and *C. Elegans*) and unassembled reads, as well as on *k*-mer document frequency tables for 29 *E. Coli* genomes. In the case of exact counts, our representation takes about a half of the space of the empirical entropy, for large enough *k*’s.

## Background

Nowadays, many bioinformatics pipelines rely on *k*-mers to perform a multitude of different tasks. Representing sequences as sets of words of length *k* generally leads to more time-efficient algorithms than relying on traditional alignments. For these reasons, alignment-free algorithms have started to replace their alignment-based counterparts in a wide range of practical applications, from sequence comparison and phylogenetic reconstruction [1–4] to finding SNPs [5, 6] and other tasks. These algorithms often require to associate some kind of information to *k*-mers involved in the analysis, that is, to build maps where keys are *k*-mers. Typical values to associate to *k*-mers are their frequencies in a particular dataset. Actual counting can be performed by one of several available *k*-mer counting tools developed in recent years [7–10]. Count tables generally include both *k*-mers and counts requiring considerable amounts of disk space to be stored. For example, the output generated by KMC [7] for a human genome, with $k = 32$ weights in at around 28GB.

In many applications, space can be significantly reduced by representing the mapping without actually storing *k*-mers. Having two independent data structures allows for more aggressive space optimizations. For example, the original sequence dataset can be used as the primary source of *k*-mers while a random-access data structure will then allow retrieving their counts efficiently. One application of such a data structure is the efficient representation of *k*-mer counts for read correction [11]. More generally, information about *k*-mer counts is increasingly used in other applications too [1, 5, 6, 12–15], which can benefit from space-efficient solutions.

*Minimal Perfect Hash Functions* (MPHFs for short) implement such an approach [16–18] and have been exstensivly used in bioinformatics in recent years [19, 20]. A MPHF bijectively maps each item from a set *S* to an index in the range $[0, |S|- 1]$. Any additional information can then be stored in an array indexed by the values returned by the MPHF. However, using an external array to store counts can be suboptimal when count values are non-uniformly distributed, i.e. the empirical entropy of their distribution is low. It is in fact known that *k*-mer counts for fully assembled genomes follow a skewed heavy-tail distribution [21, 22]. For *k* large enough, counts tend to be power-law distributed, with the majority of *k*-mers occurring only few times, mostly once. Because of this, the multiset of *k*-mer counts will typically have a fairly low empirical zero-order entropy and it could be effectively compressed to save further space. However, simply compressing the count array does not maintain queryability, which requires specialized algorithms for this task. The same considerations apply to unassembled datasets as long as the empirical entropy of the multiset of counters is low. Note also that MPHFs themselves encompass a non-negligible space overhead even without the space for storing the values, with BBHash [19] requiring around 3 bits/key whereas the theoretical minimum is 1.44.

Maps on static sets of keys can also be encoded using so-called *Static Functions* [23, 24]. Unlike MPHFs, the actual hash function and the values are encoded into the same structure. In particular, *Compressed Static Functions* (CSFs) try to benefit from the compressibility of the value array and approach the number of bits defined by the empirical entropy. This feature makes them particularly useful for representing different *k*-mer annotations, such as counts or presence information across sequences of a given sample [12–15]. CSFs can be used as readily available drop-in replacements of MPHFs since both methods assume that only *k*-mers present in the datasets can be queried for their frequency. In many cases, this is not restrictive as the “universe” of query *k*-mers can be effectively specified: for example, it can be restricted to *k*-mers from a given genome or a pan-genome. It is also conceivable to add an appropriate structure providing presence-absence information, in order to benefit from the reduction of space provided by a compact count representation.

The goal of this paper is to study data structures for storing genomic *k*-mer count tables using the smallest possible space. Our first contribution is the enhancement of CSFs with a Bloom filter to deal with datasets of very small entropy and to achieve better space usage. We call it Bloom-enhanced CSF or BCSF for short. Our second improvement takes advantage of the fact that similar *k*-mers tend to have identical (or similar) counts (see also [12]). Following this insight, we introduce a minimizer-based bucketing scheme to cluster together count values of *k*-mers with the same minimizer. A similar idea is used by some *k*-mer counting algorithms [7, 8, 25] with the difference that in our case buckets contain counts rather than the *k*-mers themselves. By choosing a representative value for each bucket, we obtain a “bucket table” that we encode using Bloom-enhanced CSF.

We study different implementation schemes based on these ideas and compare their space performance, as well as associated query time. Our results show that our algorithms are useful for both low and high entropy datasets. For large enough *k* (and large enough minimizers lengths), we are able to compress count values in *less space than their empirical entropy* while retaining fast query times. To the best of our knowledge, this is the first implementation proposing such a compact representation. We also study an extension of our algorithm to the approximate case for which we save additional space by allowing a pre-defined absolute error over queries.

## Technical preliminaries

Throughout the paper we consider a *k*-mer count table to be an associative array *f* mapping a set of *k*-mers *K*, considered static, to their counts, i.e. number of occurrences in a given dataset. $||f||_1$ stands for the L1-norm of *f*, that is $\sum _{q\in K}f(q)$.

### Minimizers

Minimizers are a popular technique used in different applications involving *k*-mer analysis. Given a *k*-mer *q* of length *k*, its minimizer of length *m*, with $m\le k$, is the smallest substring of *q* of length *m* w.r.t. some order defined on *m*-mers. The use of minimizers for biosequence analysis goes back to [26], whereas a similar concept, named *winnowing*, was earlier applied in [27] to document search. The guiding idea is that a minimizer can be considered as a “footprint” (hash value) of a corresponding *k*-mer so that similar (e.g. neighboring in the genome) *k*-mers are likely to have the same minimizer. The order of *m*-mers is usually defined via a standard non-cryptographic hash function. In this case, minimizers can be seen as a specific instance of locality-sensitive hashing, in particular of MinHash sketching [28]. The choice of hash function is not important as long as it has good statistical guarantees (randomness and uniformity). Note that the lexicographic ordering has been shown to have poor statistical properties [26].

Minimizers have been successfully applied to various data-intensive sequence analysis problems in bioinformatics, such as metagenomics (Kraken [29]) or minimizing cache misses in *k*-mer counting (KMC [7]), or mapping and assembling long single-molecule reads [30, 31]. Recently, there has been a series of works on both theoretical and practical aspects of designing efficient minimizers, see e.g. [32, 33] and references therein.

### Bloom filters

A Bloom filter is a very common probabilistic data structure that supports membership queries for a given set *S* drawn from a large universe *U*, admitting a controlled fraction of *false positives*. To insure a false positive rate $\varepsilon$, that is the probability $\varepsilon$ for an item from $U\setminus S$ to be erroneously classified as belonging to *S*, a Bloom filter *B* requires $|S|\log e\log \frac{1}{\varepsilon }$ bits, i.e. $\approx 1.44\log \frac{1}{\varepsilon }$ bits per element of *S*. For a set $T\subseteq U\setminus S$, we denote $FP_B(T)$ the set of false positives of *T*, of expected size $\varepsilon |T|$.

### Compressed static functions

A static function (SF) is a representation of a function defined on a given subset *S* of a universe *U* such that an invocation of the function on any element from *S* yields the function value, while an invocation on an element from $U\setminus S$ produces an arbitrary output. The problem has been studied in several works (see references in [23, 24]) resulting in several solutions that allow function values to be retrieved without storing elements of *S* themselves. One natural solution comes through MPHFs: one can build a MPHF for *S* and then store function values in order in a separate array. This solution, however, incurs an overhead associated with the MPHF, known to be theoretically lower-bounded by about 1.44 bits per element of *S*.

This overhead is especially unfortunate when the distribution of values is very skewed, in which case the value array may be compressed into a much smaller space. Compressed Static Functions try to solve this problem by proposing a static function representation whose size depends on the *compressed* value array. The latter is usually estimated through the zero-order empirical entropy, defined by $H_0(f)=\sum _{\ell \in L}\frac{|f^{-1}(\ell )|}{|K|}\log (\frac{|K|}{|f^{-1}(\ell )|})$, where *L* is the set of all values (i.e. $L=\{f(t)\,|\,t\in K\}\}$) and $f^{-1}(\ell )=\{t\,|\,f(t)=\ell \}$ is the set of *k*-mers with count $\ell$. $|K|\cdot H_0(f)$ can be viewed as a lower bound on the size of compressed value array, in absence of additional assumptions. Thus, the goal of CSFs is to approach the bound of $H_0(f)$ bits per element as closely as possible, in representing a static function *f*.

An overview of different algorithmic solutions for SFs and CSFs is out of scope of this paper, we refer the reader to [23, 24] and references therein. [23] proposed a solution for CSF taking an asymptotically optimal $nH_0(f)+o(nH_0(f))$ space (*n* size of the underlying value set), however the solution is rather complex and probably not suitable for practical implementation. As of today, to our knowledge, the only practical implementation of a CSF is GV3CompressedFunction [24], found in the Java package Sux4J (https://sux.di.unimi.it/). Although entropy-sensitive, the method of [24], has an intrinsic limitation of using at least 1 bit per element, due to involved coding schemes. This is a serious limitation when dealing with very skewed distributions of values, where one value occurs predominantly often and the empirical entropy can be much smaller than 1. This is precisely the case for count distributions in whole genomes, one of the applications studied in this paper.

## Methods

### Representation of low-entropy data

As mentioned earlier, Compressed Static Functions (CSF) of [24] do not properly deal with datasets generated by low-entropy distributions, in particular with entropy smaller than 1. This case occurs when datasets have a dominant value representing a large fraction (say, more than a half) of all values. This is typically the case with genomic *k*-mer count data, especially whole-genome data, where a very large fraction of *k*-mers occur just once. For example, in *E.Coli* genome ($\approx$5.5Mbp), about 97% of all distinct 15-mers occur once, with only the remaining 3% occurring more than once. For such datasets, the method of [24] does not approximate well the empirical entropy, as it cannot achieve less than 1 bit per key.

Here we propose a technique to circumvent this deficiency in order to achieve, in combination with CSFs of [24], a compression close to the empirical entropy. We start by building a Bloom filter for all *k*-mers whose value is not the dominant one, and then we construct a CSF on all positives (i.e. true and false positives) of this filter. At query time, we first check the query *k*-mer against the Bloom filter and, if the answer is positive, recover its value from the CSF.

Formally, let $K_0$ be the *k*-mers with the most common frequency. Let $|K_0|=\alpha |K|$. Assume that our Bloom filter implementation takes $C_{BF}\log {\frac{1}{\varepsilon }}$ bits per key and our CSF implementation takes $C_{CSF}$ bits per key. For the purpose of explanation, we will specify both $C_{BF}$ and $C_{CSF}$ at the end of this section.

We store keys $K\setminus K_0$ in a Bloom filter *B* and build a CSF for $(K\setminus K_0)\cup FP_B(K_0)$. The total space is

1 $$\begin{aligned} C_{BF}(1-\alpha )|K|\log {\frac{1}{\varepsilon }}+C_{CSF}|K|((1-\alpha )+\varepsilon \alpha ). \end{aligned}$$

The Bloom filter enables space saving only if $\alpha$ is sufficiently large. To decide if we need a Bloom filter, we have to verify if the inequality

2 $$\begin{aligned} C_{BF}(1-\alpha )|K|\log {\frac{1}{\varepsilon }}+C_{CSF}|K|((1-\alpha )+\varepsilon \alpha ) < C_{CSF}|K|. \end{aligned}$$

holds for some $\varepsilon <1$. Note again that $C_{CSF}$ on the left and right sides are not exactly the same in reality, however assuming them the same is not reductive because of specificities of the CSF implementation we use. We will elaborate further on this later on. Then (2) rewrites to

3 $$\begin{aligned} \frac{C_{BF}}{C_{CSF}} \frac{1-\alpha }{\alpha }\log {\frac{1}{\varepsilon }}+\varepsilon < 1. \end{aligned}$$

Using simple calculus, we obtain that if $\frac{C_{BF}}{C_{CSF}} \frac{1-\alpha }{\alpha }>\ln 2$ (that is, $\frac{C_{BF}}{C_{CSF}} \frac{1-\alpha }{\alpha }\log e>1$), then (3) never holds for $0<\varepsilon <1$. The left-hand side of (3) reaches its minimum for

4 $$\begin{aligned} \varepsilon _0 = \frac{C_{BF}}{C_{CSF}} \frac{1-\alpha }{\alpha }\log e, \end{aligned}$$

and this minimum is smaller than 1 if $\varepsilon _0<1$. We conclude that in order to decide if a Bloom filter enables space saving, we have to check the value $\varepsilon _0$. If $\varepsilon _0\ge 1$, we do not need a Bloom filter, otherwise we need one with $\varepsilon =\varepsilon _0$. This shows that a Bloom filter is needed whenever

5 $$\begin{aligned} \alpha > \frac{C_{BF}\log e}{C_{CSF}+ C_{BF}\log e} \end{aligned}$$

For $C_{BF}= C_{CSF}$, this gives $\alpha > 0.59$.

In order to apply equation (4), we need estimates of $C_{BF}$ and $C_{CSF}$, that is, estimates of the number of bits per element taken by our implementations of Bloom filter and CSF. For $C_{BF}$, we have $C_{BF}=1.44$ corresponding to the theoretical coefficient of Bloom filters. On the other hand, we experimentally estimated $C_{CSF}$ associated with the implementation we use as a function of the empirical entropy $H_0$, giving:

6 $$\begin{aligned} C_{CSF}= {\left\{ \begin{array}{ll} 0.22 H_0^2 + 0.18 H_0 + 1.16,&{} \text {if } H_0 < 2\\ 1.1 H_0 + 0.2,&{} \text {otherwise}. \end{array}\right. } \end{aligned}$$

In the rest of the paper we use the term *Bloom-enhanced Compressed Static Function*, BCSF for short, to speak about CSF possibly augmented by a prior Bloom filter, as described in this section. Algorithm 1 summarizes the computation of the BCSF data structure.

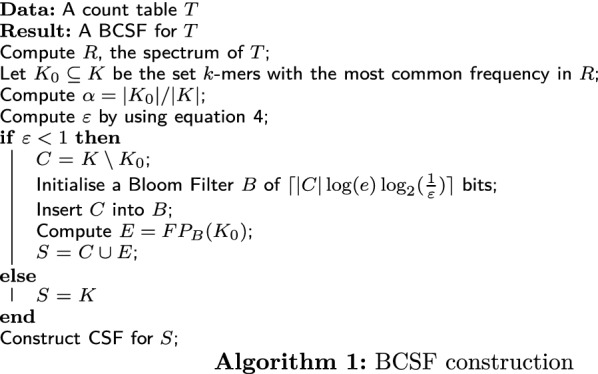



### Minimizer bucketing

A key idea to reduce the computational burden of counting *k*-mers, is to use minimizers to bucket *k*-mers and split the counting process across multiple tables (cf e.g. [7]). Here we use the same principle to bucket count values instead of *k*-mers themselves. Let $M_m(K)=\{\mu _m(q)\,|\,q\in K\}$ be the set of minimizers of all *k*-mers of *K* of a given length $m<k$. We map the input set *K* onto the (smaller) set $M_m(K)$. To each minimizer $s\in M_m(K)$, corresponds the bucket $\{f(q)\,|\,q\in K, \mu _m(q)=s\}$. We call a minimizer and the corresponding bucket *ambiguous* if this set contains more than one value. The guiding idea is to replace *f* by a mapping *g* of $M_m(K)$ to ${\mathbf {N}}$. Querying value *f*(*q*) for a *k*-mer $q\in K$ will reduce to first querying $g({\mu }_m(q))$ and then possibly “correcting” the retrieved value. In other words, for each bucket, we replace its set of counts with one representative value and we split the query into two operations: retrieving the representative from the buckets and correcting to reconstruct the original value. The rationale is that *k*-mers having the same minimizer tend to have the same count allowing multiple values to be dealt with by a single bucket. We consider two implementations which differ on how the representatives are chosen and how corrections are applied.

Our *first implementation* is named AMB (from AMBiguity). An extended version of AMB (explained below) is presented in Algorithm 3. For non-ambiguous minimizers *u*, AMB defines *g*(*u*) to be the unique value of the bucket. For ambiguous minimizers *v*, we set $g(v)=0$, where 0 is viewed as a special value marking ambiguous buckets (*k*-mers with count 0 are not present in the input). This has the disadvantage of providing no information about the values of ambiguous buckets, and also of making *g* less compressible (because of an additional value). On the other hand, this has the advantage of distinguishing between ambiguous and non-ambiguous buckets and allows the query to immediately return the answer for *k*-mers hashing to non-ambiguous buckets. As a consequence, unambiguous *k*-mers are not propagated to the second layer, and if $g(\mu _m(q)) \ne 0$ it can be immediately returned as *f*(*q*). We then have to store mapping *f* restricted only to *k*-mers from ambiguous buckets, which we denote ${\tilde{f}}$. Both mappings *g* and ${\tilde{f}}$ are stored using BCSFs.

Our *second implementation*, named FIL (from FILtration), is shown in Algorithm 2. Here, *g*(*s*) is defined to be the majority value among all values of its bucket, ties resolved arbitrarily. In particular, if *s* is a non-ambiguous minimizer then *g*(*s*) is set to the unique value of the bucket. In practice, computing the majority value may incur a computational overhead as this requires storing bucket values until all values are known. An option to cope with this, not explored further in this work, is to use the “approximate majority” computed by the online Boyer-Moore majority algorithm [34]. We then store a “correcting mapping” $h:K\rightarrow {\mathbf {N}}$ defined by $h(q)=f(q)-g(\mu _m(q))$. That is, we construct another counting table *h* where each *k*-mer is associated to the correction factor *h*(*q*), which, added to the representative *g*(*s*) results in the original count *c*. Both mappings *g* and *h* are stored using BCSFs. The rationale for this scheme is that, due to the properties of minimizers, *h*(*q*) is supposed to be often 0, which makes *h* well compressible using BCSF. Note that because of the majority rule, 0 will always be the majority value of *h*. Therefore, the Bloom filter of the BCSF storing *h* (if any) will hold *k*-mers *q* with $f(q)\ne g(\mu _m(q))$ (i.e. $h(q)\ne 0$). Then the BCSF will store *h* restricted to *k*-mers with $h(q)\ne 0$ together with a subset of *k*-mers (false positives of the Bloom filter) for which $h(q)=0$.

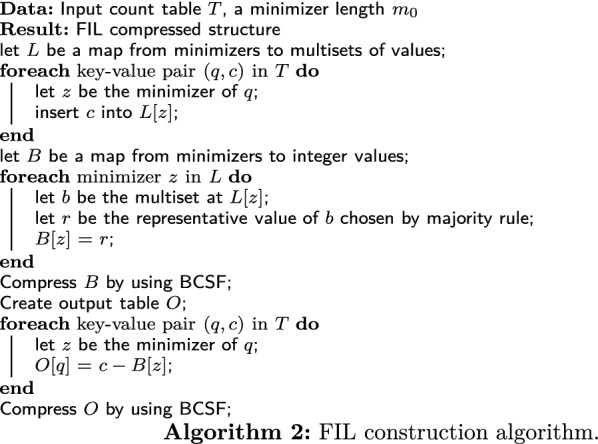



### Cascading

An intermediate layer corresponding to a minimizer length $m<k$, introduced in Sect. 3.2, can be viewed as a “filter” providing values for some *k*-mers and “propagating” the other *k*-mers to the next layer. Therefore, both implementations can be cascaded into more than one layer. This construction is reminiscent of the BBHash algorithm [19] or to cascading Bloom filters from [35].

For $m_1< m_2 <... m_\ell \le k$, each layer *i* is then input some map $f_{i-1}$ defined on a subset of *k*-mers $K_{i-1}\subseteq K$ ($f_0=f$, $K_0=K$) and outputs another map $f_i$ defined on a smaller subset $K_i\subseteq K_{i-1}$. Each layer stores a bucket table for minimizers $M_{m_i}(K)=\{\mu _{m_i}(q)\,|\,q\in K_{i-1}\}$. The specific definition of $f_i$ and $K_i$ depends on the implementation.

The multi-layer scheme is particularly intuitive for the AMB implementation, where each layer stores a unique value for non-ambiguous minimizers and a special value 0 otherwise. In this case, $K_i$ consists of those *k*-mers of $K_{i-1}$ hashed to ambiguous buckets, and $f_i$ is simply a restriction of *f* to those *k*-mers. Algorithm 3 shows a pseudo-code of multi-level AMB extended to the approximate case (see Sect. 3.4 below). The multi-layer version of the FIL scheme is shown in Appendix (Algorithm 4).

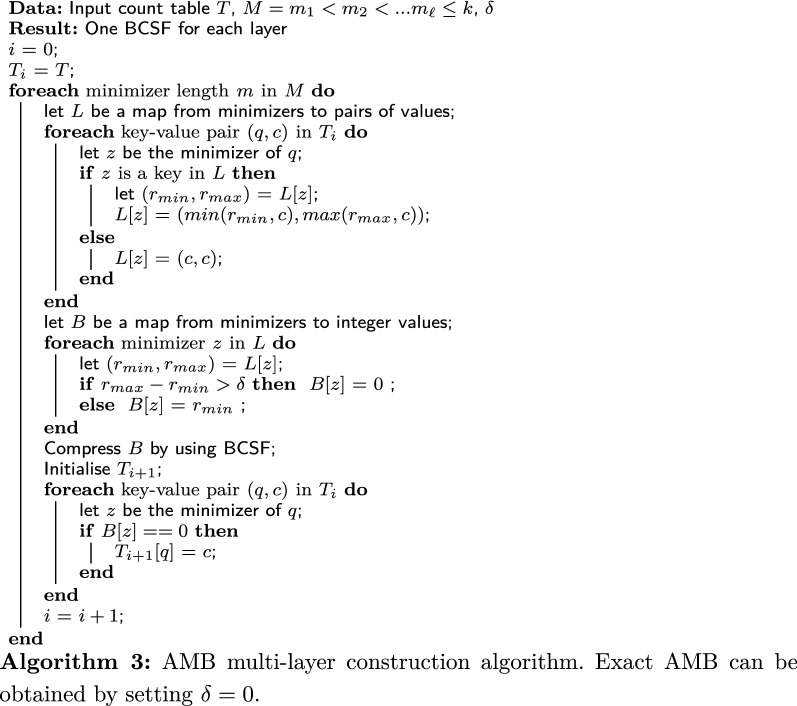



### Extension to approximate counts

In addition to cascading, AMB can also be easily extended to work as an approximation algorithm. Consider, to this end, the layered bucketing procedure desribed in 3.3. In the exact case, a bucket is marked as colliding whenever it contains two or more distinct count values. In the approximate case, a collision is defined if a bucket contains a pair of counts, $c_i$, $c_j$ such that $|c_i - c_j|> \delta$ with $\delta$ a pre-defined maximum absolute error. With this modification, the algorithm guarantees to output a value within the absolute error $\delta$ from the true count.

We chose *g*(*s*) to be the minimum value in a bucket if the bucket is unambiguous. The rationale of using minimum is the decreasing behavior of *k*-mer spectra which implies that smaller counts are more frequent and therefore more likely to constitute the majority. In order to detect collisions, it is then sufficient to only remember the maximum *max*(*s*) and minimum *min*(*s*) values seen by each bucket and check if $max(s) - min(s) > \delta$. If that is the case, then the bucket is marked as colliding, otherwise *min*(*s*) is chosen as representative (see Algorithm 3).

## Results and discussion

Three datasets were used in this study:

1. The collection of fully assembled *Escherichia Coli* genomes from [2], from now on referred to as “df”.
2. *Escherichia Coli* Sakai strain (NCBI accession number B000007) from the previous collection [2] but from now on referred to as “Sakai” to highlight its stand-alone usage.
3. Full reference genome of *Caenorhabditis Elegans*, strain *Bristol N2* downloaded from RefSeq (accession number GCF_000002985.6). We will refer to this dataset as “Elegans”.
4. “SRR10211353” run of Illumina reads (10x coverage, *Escherichia Coli*) downloaded from NCBI SRA (accession number SAMN12880992).

Unless stated otherwise, FIL and AMB were run on all possible combinations of two and three minimizer lengths for $k \in [13, 15, 18, 21]$ with only the best combinations reported using the following naming convention:

- CSF: baseline CSF implementation from Sux4J [24].
- BCSF: extended CSF with Bloom filter from Sect. 3.1. It may get reduced to a simple CSF if the Bloom filter is not useful.
- AMB $m_1$ *k*: our *first implementation*, selecting each representative by minimum and marking colliding buckets with a special value.
- AMB $m_1$ $m_2$ *k*: same as before but with an additional layer.
- FIL $m_1$ *k*: our *second implementation*, saving into each bucket a majority-selected representative and saving corrections into its second layer.
- FIL $m_1$ $m_2$ *k*: same as before but with an additional layer.

### Compression of skewed data

**Fig. 1:**
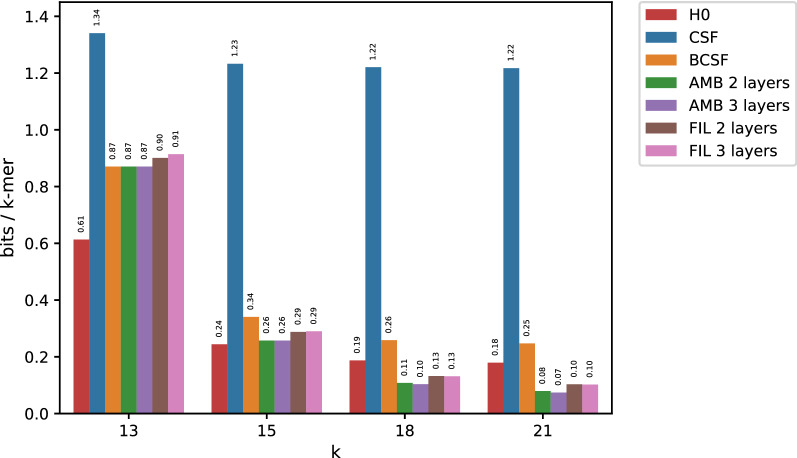
Results for the Sakai dataset for big values of *k*. For presentation purposes, H0 is represented as an additional red column in each subgroup

Figure 1 reports memory usage when compressing the Sakai dataset. Simple CSF use more than 1 bit/*k*-mer, while Bloom-enhanced CSF (BCSF) is considerably more efficient, reaching space closer to the entropy. For relatively small *k*’s ($k=13$) AMB and FIL give almost the same results as BCSF, that is, minimizer-based bucketing is not helpful. For larger *k*’s, however, both AMB and FIL lead to significant space reductions, eventually breaking the entropy barrier for larger values of *k* ($k=18,21$). This demonstrates that for larger *k*’s, minimizers provide an effective way of factoring the space of *k*-mers in such a way that *k*-mers with equal counts tend to have the same minimizer.

More in detail, for larger *k*, the overwhelming majority of buckets are unambiguous (e.g. more than $99\%$ of them, for $k=18, m=13$). As a consequence, AMB is able to “filter out” a very large number of *k*-mers with few buckets. Only a small set of *k*-mers, corresponding to ambiguous buckets, are propagated to the next layer. This, combined with the prevalence of one value due to the skewedness of the count distribution, and the fact of using minimizers with increasing lengths, leads to highly compressible bucket tables. Altogether, this enables breaking the empirical entropy lower bound.

The situation is similar for FIL: its first layer is even better compressible than the one of AMB, due to the absence of the additional special value which makes the table of AMB slightly less compressible. On the other hand, the BCSF of the second layer table of FIL turns out to take more space than that of AMB. This is because its Bloom filter operates on the large set of all *k*-mers, which implies a very small value of $\varepsilon$ to keep the set of false positives under control, and as a consequence, a relatively large Bloom filter. Overall, FIL turns out to yield a slightly larger space than AMB.

For small *k*’s, none of our methods beats the empirical entropy, with minimizers unable to provide an efficient mean to factor the space of *k*-mers according to count values. On the contrary, we observe that in this case applying a BCSF to the input table provides the most efficient solution.

**Fig. 2:**
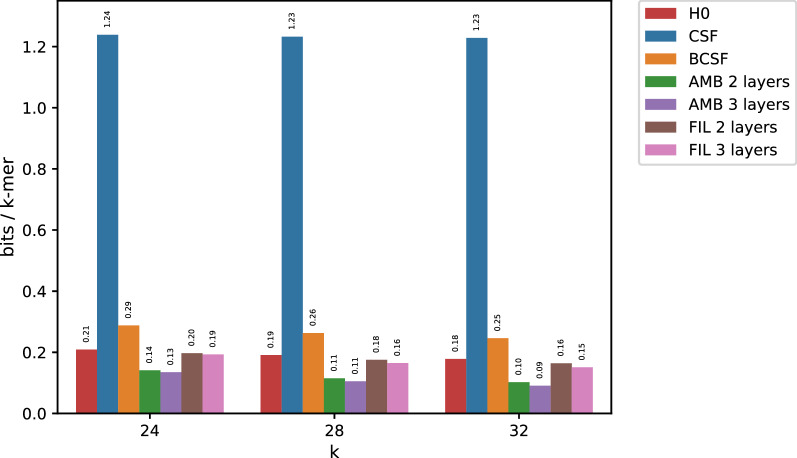
Results when compressing the Elegans dataset

Since longer *k*-mers lead to more skewed data, and by extension, to smaller entropies, both AMB and FIL better compress whole genome count tables for increasing *k*s. To test this assumption we chose to compress the Elegans dataset (around 100 Mbp). We randomly chose $m_1=18$ and $m_2=19$ for both three-layer AMB and FIL (ignoring $m_2$ for the two layered versions). Figure 2 demonstrates that our algorithms are not limited to bacterial genomes. Instead they are applicable in the general case as long as count tables are computed on fully assembled data and *k* is large enough. Note that, under such a regime, larger values of *k* only reduce the entropy of the data, leading to more succinct representations whereas simple CSF could not go below 1.2 bits/*k*-mer.

### Compression of higher entropy data

**Fig. 3:**
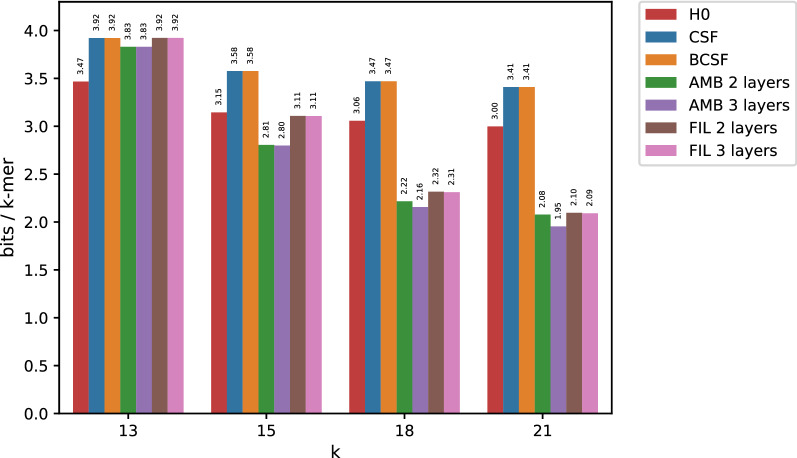
Compressed space usage for the high entropy SRR10211353 dataset

With very skewed data, collisions of *k*-mer counts may happen between unrelated *k*-mers simply because one counter value strongly dominates the spectrum. In order to demonstrate the utility of minimizers in a more general setting other than whole genome count tables, we applied our methods to less skewed distributions. To this end, we compressed the *k*-mer count tables when using dataset SRR10211353 whose results are presented in Fig. 3. As opposed to fully assembled genomes, entropy in this case remains well above 1 even for larger values of *k*. Nonetheless, both AMB and FIL are able to produce representations more compact than both simple CSFs and BCSFs for all $k > 13$, beating the entropy lower bound.

**Fig. 4:**
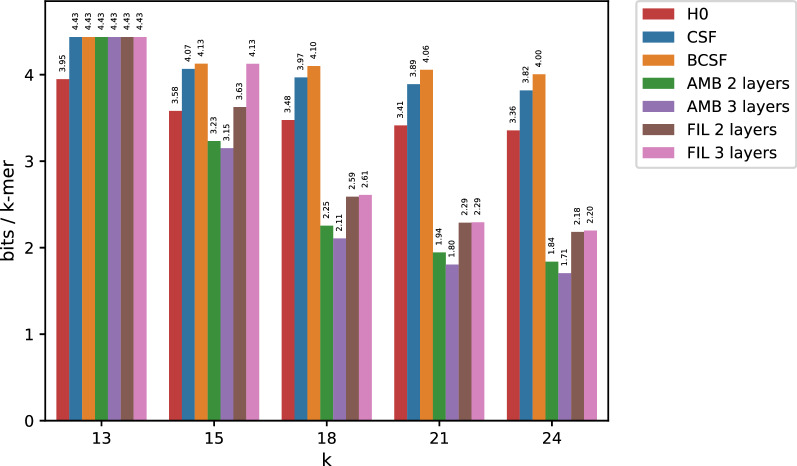
Compressed space usage for the high entropy df dataset

Further proof of the ability of minimizer-based bucketing to boost compression of *k*-mer count tables can be found in Fig. 4. Here, we compressed the table produced by counting the number of occurrences for each *k*-mer among the 29 *E. coli* genomes of dataset df (note that df is a mnemonic for “document frequency”). Note that entropy does not decrease as rapidly as before with increasing *k*, despite counts bounded in the range [1, 29].

The use of minimizers for larger *k*’s, proves to be beneficial again, with AMB and FIL requiring much less space than the empirical entropy of the data. Again, when $k=13$, both AMB and FIL do not have an advantage over a simpler (B)CSF. For even smaller *k*-mers (B)CSF remains the best option (see Additional Fig. 5). The seemingly erroneous exceptions (BCSF taking more space than simple CSF) are explained by the approximation carried out by formula (2) (assumption of equal values of $C_{CSF}$ in both sides).

**Fig. 5:**
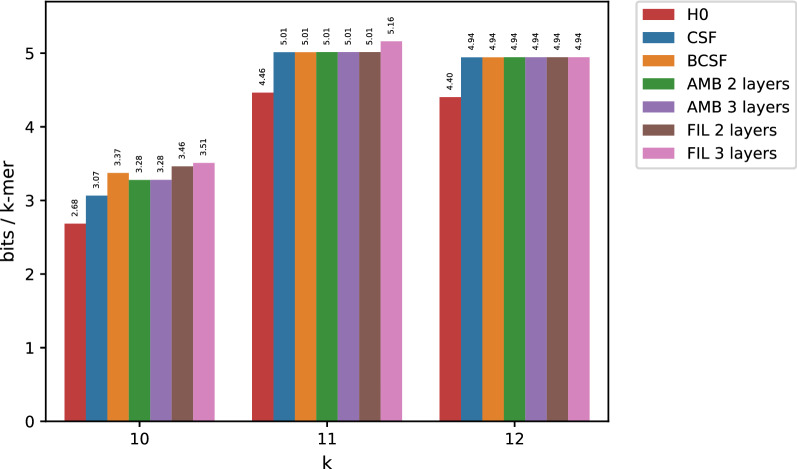
Compressed space usage for the high entropy df dataset when using small values of *k*

### Approximate counts

**Fig. 6:**
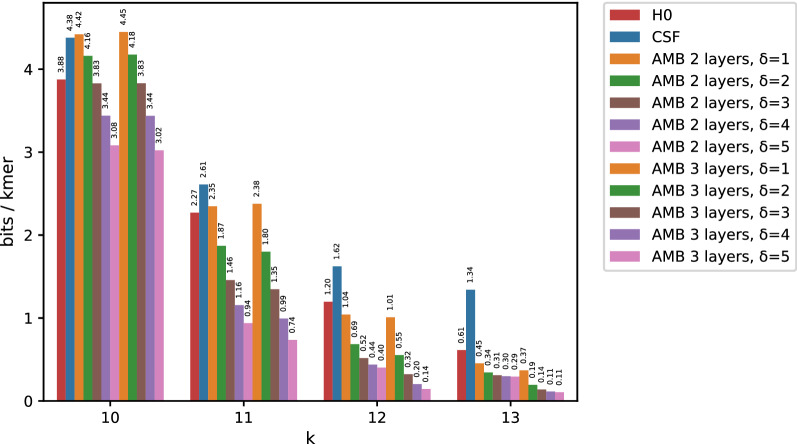
Space usage when using the approximated version of AMB. Entropy (red columns) and CSF (blue columns) are reported for comparison. Unlike Fig. 7, AMB is able to break the empirical entropy lower bound when small errors are acceptable

**Fig. 7:**
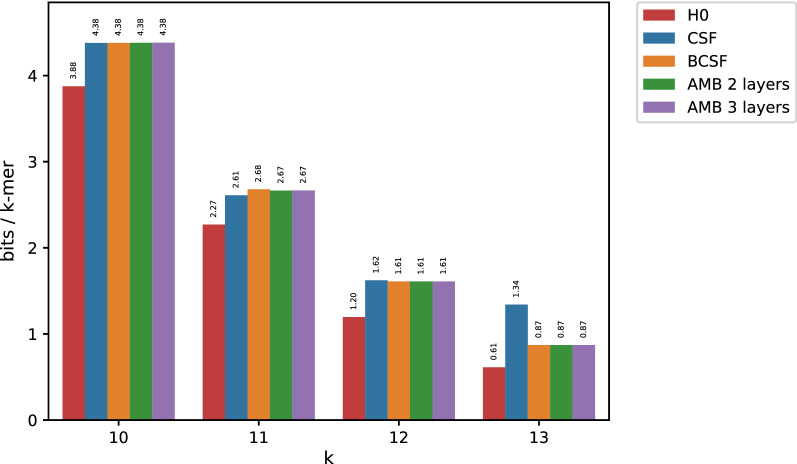
Space usage of AMB for the Sakai dataset with small *k* (FIL is slightly worse and was omitted)

In many applications, it is acceptable to tolerate a small absolute error in retrieved counts. Figure 6 reports space usage when using the approximate version of AMB ($\delta > 0$, see section 3.4) on the Sakai dataset. Results for the exact algorithm ($\delta = 0$) are reported in Fig. 7 for comparison.

In order to show how the approximate algorithm achieves better compression ratios, *k* was chosen from [10, 11, 12, 13], a range of values which is particularly difficult for AMB (or FIL) with $\delta =0$. Trying all possible minimizer combinations compatible with such *k*s, the best results are obtained for very short minimizer lengths (between 1 and 5). Building minimizer layers for such small values of *m* does not lead to better compression than simple (B)CSFs, with Fig. 7 showing no tangible differences between (B)CSFs and AMB (or FIL). For these reasons, minimizer lengths in Fig. 6 are equal to $k-1$ (and $k-2$) for every choice of *k* (e.g. if $k=10$, layers will be 8, 9, 10 for three-layer AMB). Using the same small lengths of the exact case would not allow meaningful bucketing of counts values.

An interesting observation about the approximate case is that AMB with three layers is substantially better than AMB with two layers only for $k=12$ and $k=13$. For $k=10$ and $k=11$ both versions give almost the same results.

### Query speed

**Fig. 8:**
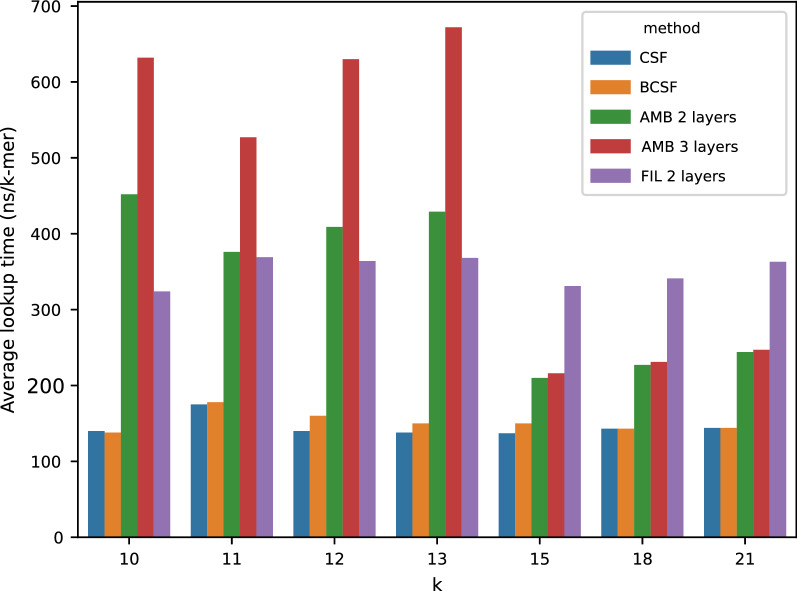
Average query time for AMB with 2 and 3 layers and FIL with 2 layers

Figure 8 shows query time averaged over all distinct *k*-mers, in ns/*k*-mer. Simple CSFs, not surprisingly, are the fastest method, with BCSF having a negligible effect on the average query speed. On the other hand, bucketing has a tangible effect on performance, with speed negatively affected by additional layers. For short *k*-mers, both FIL and AMB are slower than the simple CSF by a factor equal to their number of layers.

The situation is different for larger *k*’s where AMB is only marginally slower than a bare-bones CSF. This is because most queries are solved without accessing all layers every time, thanks to unambiguous buckets. Two-layered FIL, on the other hand, gives almost constant average query times across all test, since all queries have to access both of its layers to reconstruct the exact count value. We did not perform tests for FIL with 3 layers because it will always be slower than the two layered version.

### Choosing minimizer lengths

In all reported cases, good minimizer lengths for the first layer ($m_0$) follow the rule: $m_0 > m_s = (\log _4 |G|+ 2)$ with $|G|$, the size in base pairs of the genome. Smaller $m_0$, are no longer capable of partitioning *k*-mers in a meaningful way. Furthermore, space tends to first monotonically decrease to a minimum for increasing minimizer lengths, to increase again once the optimal value is passed. It is therefore possible to find the minimum by sequentially trying all possible minimizers greater than $m_s$ and stop as soon as the compressed size starts to increase again.

If it is not possible to choose $m_0 > m_s = (\log _4 |G|+ 2)$ because, e.g. *k* is already too small, approximation might be a viable option even for relatively small $\delta$. The only caveat to pay attention to in this case is to check if a minimizer layer would be useful or not. If yes, $\delta$ can be incremented without further adjustments compared to exact case. If not, minimizer lengths for the bucketing layers should be chosen as big as possible to allow meaningful bucketing of count values.

Our results also show how multiple layers have a marginal effect on final compression sizes. In case of AMB, using three layers is always helpful, compared to the two-layer case. Best results are usually achieved for combinations including the best minimizer length obtained for the two-layer case. On the other hand, FIL with three layers seems to be advantageous only for low entropy data, performing worse than its two-layer counterpart when compressing document frequency tables and for small *k*’s.

## Conclusions

In this work, we introduced three data structures to represent compressed *k*-mer count tables. Our BCSF algorithm combines Compressed Static Functions, as implemented in Sux4J software [24], with Bloom filters. This allows for a much better compression for skewed distributions with empirical entropy smaller than 1. Note that, to the best of our knowledge, this is the first time CSFs are used in a bioinformatics application. We also provide a method to dimension the Bloom filter in a BCSF in order to minimise the final space. Our two other algorithms, AMB and FIL, pair BCSF with a bucketing procedure where count values are mapped into buckets according to minimizer values of respective *k*-mers. This locality-sensitive hashing scheme allows us to efficiently factor the space of counts, which leads to breaking the empirical entropy lower bound for large enough *k*’s. AMB and FIL use slightly different strategies in decomposing the input table across minimizer layers. Our last contribution is an extension of AMB to the approximate case, gaining more space at the expense of a small and user-definable absolute error on the retrieved counts.

We validated our algorithms on four different types of count tables, two fully assembled genomes (*E.Coli* and *C.Elegans*) of different sizes, one dataset of *E.Coli* reads at 10x coverage and one document frequency table of 29 different *E.Coli* genomes, for different *k*-mer lengths showing how BCSF, AMB and FIL behave in different situations. AMB and FIL have a clear advantage when minimizers are long enough to bucket *k*-mers in a meaningful way, for both skewed and high entropy data. When it is not possible to define a long-enough minimizer length, the advantage of using intermediate minimizer layers vanishes, and simple CSF and its BCSF provide a better solution.

At query time, CSF and BCSF are the fastest methods requiring about 100ns on average for a single query. For a fixed number of layers, AMB is faster than FIL in all situations when minimizers are useful. FIL becomes faster than AMB only for those cases when both algorithms achieve worse compression ratios than simple (B)CSF.

We consider this study to be the first step towards designing efficient representations for *k*-mer count tables occurring in data-intensive bioinformatics applications. One possible future direction is compression of RNA-Seq experiments where counts may translate expression levels of genes. Another example is metagenomics where different species may be present with different abundances which can be captured by *k*-mer counts. In such applications, efficient representation of *k*-mer counts can be particularly beneficial.

## Data Availability

*Datasets* $\bullet$ Collection of 29 fully assembled *Escherichia Coli* genomes from [2]. Approximately 25 million *k*-mers $\bullet$ Full genome of *Caenorhabditis Elegans*, strain *Bristol N2* downloaded from RefSeq (accession number GCF_000002985.6). $\bullet$ SRR10211353 run of Illumina reads (10x coverage, *Escherichia Coli*) downloaded from NCBI SRA (accession number SAMN12880992). In one of our experiments we specifically targeted the *Sakai* strain of *E.Coli* (one of the genomes included in [2]) with NCBI accession number B000007. *Implementation* All construction code is written in python, except for the CSF part which is handled by a simple Java program using Sux4J [24]. An utility written in C using the code provided by Sux4J for reading and querying its CSFs provides time measurements. We use xxHash https://github.com/Cyan4973/xxHash to define an ordering over minimizers. All our code is available at https://github.com/yhhshb/locom.git. *Experimental setup* Experiments were performed on a machine equipped with an Intel$^\text{\textregistered }$ Core$^{\mathrm{TM}}$ i7-4770k (Haswell), 8 GB of RAM and Kubuntu 18.04.

## Appendix

Multilayer FIL algorithm
